# Prediction Network of Metamaterial with Split Ring Resonator Based on Deep Learning

**DOI:** 10.1186/s11671-020-03319-8

**Published:** 2020-04-15

**Authors:** Zheyu Hou, Tingting Tang, Jian Shen, Chaoyang Li, Fuyu Li

**Affiliations:** 1 Hainan University, No. 58, Renmin Avenue, Haikou, 570228 Hainan Province China; 2 Chengdu University of Information Technology, Chengdu, 610225 China; 3 Dongguan ROE Technology Co., Ltd., Dongguan, 523000 China

**Keywords:** Deep learning, Split ring resonator, Metamaterial

## Abstract

The introduction of “metamaterials” has had a profound impact on several fields, including electromagnetics. Designing a metamaterial’s structure on demand, however, is still an extremely time-consuming process. As an efficient machine learning method, deep learning has been widely used for data classification and regression in recent years and in fact shown good generalization performance. We have built a deep neural network for on-demand design. With the required reflectance as input, the parameters of the structure are automatically calculated and then output to achieve the purpose of designing on demand. Our network has achieved low mean square errors (MSE), with MSE of 0.005 on both the training and test sets. The results indicate that using deep learning to train the data, the trained model can more accurately guide the design of the structure, thereby speeding up the design process. Compared with the traditional design process, using deep learning to guide the design of metamaterials can achieve faster, more accurate, and more convenient purposes.

## Introduction

Nano-optics is an interdisciplinary subject of nanotechnology and optics. In recent years, by constantly designing structures with different sub-wavelength sizes in order to achieve special interactions with incident light, scientists have succeeded in manipulating certain transmission characteristics of light [1–3]. Since metamaterials were proposed, they have attracted the attention of many scholars in this field, and concurrently their related theoretical study [4, 5], process [6, 7], and applied [8] research are all advancing at the same speed. Many peculiar functions have been realized, including holographic imaging, perfect absorption [9], and flat lenses [10]. Due to the rapid development of terahertz technology and its unique characteristics, it has also become a popular research topic in the field of metamaterials in recent years [11–13].

Although the application of metamaterials is very wide, the traditional design method requires the designer to repeatedly perform complex numerical calculations on the structure being designed. This process consumes huge time and computing resources. Therefore, it is urgent to find new ways to simplify or even replace traditional design methods.

As a cross-disciplinary field, machine learning covers many disciplines including life sciences, computer sciences, and psychology, it has been working to use computers to imitate and implement human learning processes to acquire new knowledge or skills. The basic principle of machine learning can be simply described as the use of computer algorithms to obtain the correlation among a large amount of data or to predict the rules among similar data and finally achieve the purpose of classification or regression. Until now, many machine learning algorithms have been applied to the designation of metamaterials and have achieved significant results, including genetic algorithms [14], linear regression algorithms [15], and shallow neural networks. As the structure turns more and more complex and the changes in the structure become more diverse, problems will require more time to solve. At the same time, the highly nonlinear nature of the problems makes it difficult for simple machine learning algorithms to obtain accurate predictions. In addition, to design a matching metamaterial structure for a specific electromagnetic effect requires designers to try and perform complex numerical calculations on the structure. These processes will consume a tremendous amount of time and computing resources.

As one of the most outstanding algorithms in the field of machine learning, deep learning has made world-renowned achievements in various related fields such as computer vision [16], feature extraction [17], and natural language processing [18]. At the same time, successes in other non-computer related fields are numerous, including many basic disciplines such as life sciences, chemistry [19], and physics [20] [21]. Therefore, applying deep learning to the design of metamaterials is also a hot research direction at present, and many outstanding works have appeared [22–24].

Inspired by deep learning, this paper reports a study using a machine learning algorithm based on a deep neural network to predict the structure of the split-ring resonator (SRR) to achieve the purpose of designing on demand. In addition, the forward network and the reverse network are innovatively trained separately, which not only can improve the accuracy of the network, but also can achieve different functions through flexible combination. The results show that the method can achieve MSE of 0.0058 and 0.0055 on the training set and validation set, respectively, and displays good robustness and generalization. With the trained model guiding the design of metamaterial structures, the design cycle can be shortened to days or even hours, and the improvement in efficiency is obvious. In addition, this method also has good scalability and only needs to change the training set data to design different inputs or different structures on demand.

## Theory and method

### COMSOL model building

**Fig. 1:**
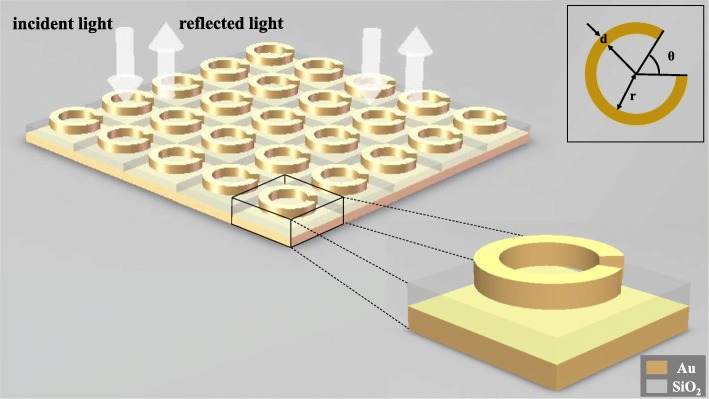
Schematic diagram of the structure. The entire metasurface is composed of meta-atoms arranged repeatedly in two directions, and linearly polarized light is incident perpendicular to the metasurface. Each meta-atoms is composed of a gold ring, a silica bottom, and a gold bottom in order from top to bottom. The uppermost gold ring contains three structural parameters, namely the line width *d*, the opening angle *θ*, and the inner ring radius *r*

In order to show that deep learning can be applied to the reverse design of metamaterials structures, we modeled a three-layer SRR structure consisting of a gold ring, a silica bottom, and a gold bottom to observe its electromagnetic response under the action of the incident light. As shown in Fig. 1, the opening angle *θ* of the gold ring, the inner radius *r* of the ring, and the line width *d* of the ring are selected as independent variables of this structure. When a beam of linearly polarized light enters the metamaterials normally, the wavelength-reflectance curves under different structures are obtained by changing the structural variables. The thickness of the Au ring is 30 nm, of the bottom of SiO_2_ is 100 nm, and of the bottom of Au is 50 nm, and the size of the meta-atoms is 200 nm by 200 nm.

Use COMSOL Multiphysics 5.4 [25] for modeling, choose three-dimensional space dimension, choose optics ≥ wave optics ≥ electromagnetic wave frequency domain (ewfd) for the physical field and select the wavelength domain for research. Create the above model in geometry. The material of each part and its refractive index are defined in order in the material, and ports and periodic conditions are added in the electromagnetic wave frequency domain.

### Building a deep learning neural network model

We have constructed a reverse network and a forward network for the metamaterial structure. The reverse network can predict the structural parameters of the SRR from the given two sets of wavelength-reflectance curves with different polarization directions. The forward network can predict the wavelength-reflectance curves in two polarization directions by the given structural parameters. The function of the reverse network is the main body of the prediction function. The role of the forward network is to verify the prediction results of the reverse network to observe whether the prediction results meet the required electromagnetic response.

Use eclipse 2019 as the development platform, python3.7 as the programming language, and TensorFlow 1.12.0 as the development framework.

The two networks are trained separately to keep the training results of each network from being affected by the error of the other network, which thereby ensures the respective accuracy of the two networks.

**Fig. 2:**
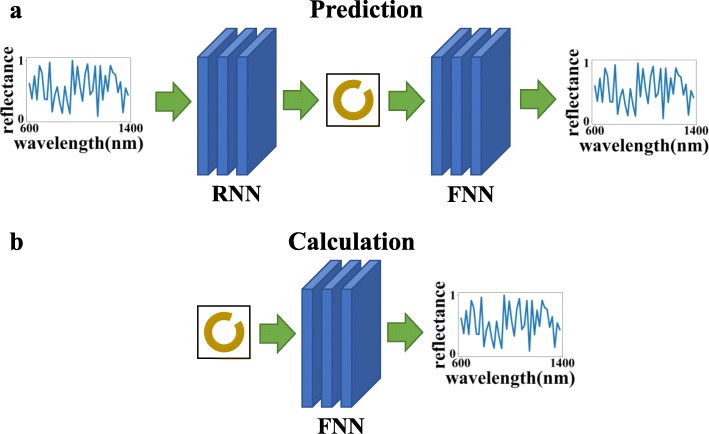
In this figure, FNN refers to the forward neural network, and RNN refers to the reverse neural network. The top graph (**a**) indicates that the two networks can be connected to achieve the effect of prediction and verification, and the bottom graph (**b**) indicates that the forward response network alone can be used to calculate the optical response

As shown in Fig. 2, another advantage of training the two networks separately is that they can be used for different purposes through different connection sequences: (a) reverse network + forward network, which can use the given wavelength-reflectance curve to calculate the structure parameters, make predictions and verify whether the prediction results meet the needs, and (b) using the forward network alone can simplify the calculation process of the numerical calculation method and reduce the calculation time.

It is worth noting that the process of inputting and obtaining the results of the trained model using the method of deep learning takes an extremely short time. And whenever new data is obtained through simulation or experiment, the model can be used for further training. Studies have shown that with the continuous increase of training data, the accuracy of the model will become higher and higher, and the generalization performance better and better [26].

The parameters of the structure are multiple sets of continuous eigenvalues, which belong to the regression problem. In recent years, fully connected networks have been the focus of deep learning networks on regression issues and shown the characteristics of high reliability, large data throughput, and low latency. Making some adjustments on a fully connected network will allow the network to better predict the structure.

**Fig. 3:**
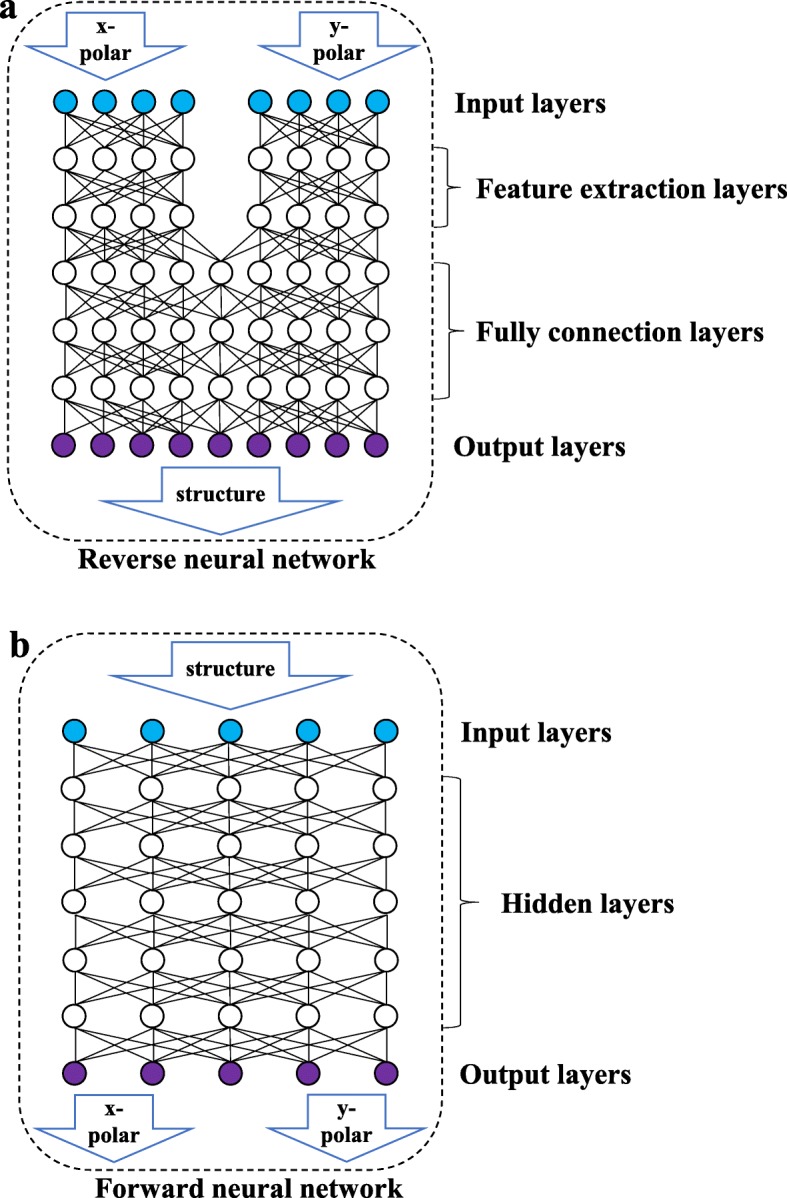
Schematic diagram of the network structure. The above figure shows the reverse network. The reverse network consists of an input layer, a feature extraction layer, a fully connected layer, and an output layer. The following figure shows the forward network, which consists of an input layer, a hidden layer, and an output layer

As shown in Fig. 3b, the forward network is a fully connected network in which all nodes of the two adjacent layers are connected to one another. The input data is the structural parameter, and the output is the wavelength-reflectance curve of the two polarization directions. As shown in Fig. 3a, the reverse network consists of a feature extraction layer (FE layer) and a fully connected layer (FC layer). The FE layer includes two sets of fully connected networks which are not connected to each other and processes the wavelength-reflectance curves of the linearly polarized light in the two directions to extract some features of the input data. The FC layer will learn the extracted features and output the structural parameters. Because of the characteristics of high cohesion and low coupling between the wavelength-reflectance curves in different polarization states, separating the inputs of two sets of polarized light data in different directions can prevent the network from being disturbed by data standardization during the data extraction process. The forward network does not involve multiple sets of inputs and does not need to consider mutual interference between data, so it does not have a feature extraction layer.

**Fig. 4:**
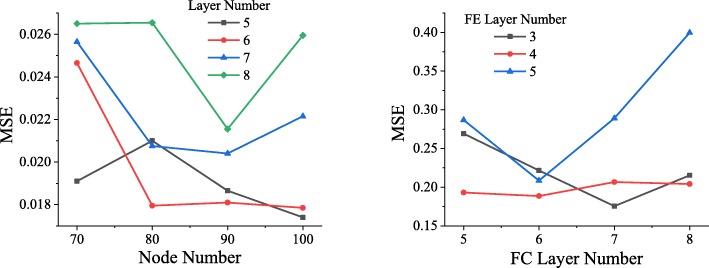
Comparison of network structures. In the figure on the left, the horizontal axis represents the number of nodes in each layer, the vertical axis represents MSE, and the black, red, blue, and green represent the situation when the hidden layer contains 5, 6, 7, and 8 layers, respectively. In the figure on the right, the horizontal axis indicates the number of layers in the fully connected layer, the vertical axis indicates MSE, and the black, red, and blue lines indicate the situation when the FE layer includes 3, 4, and 5, respectively

In order to determine the optimal network structure, networks in different structures are trained using the same set of data. As shown in Fig. 4, after the data has experienced 50 epochs (when all the data has undergone a complete training, it is called an epoch), the MSE reached by the forward network of different structures. As can be seen from the left picture of Fig. 4, when the forward network contains 5 hidden layers, each layer containing 100 nodes, the lowest MSE achieved is about 0.0174, so the forward network of this structure will be selected.

Similarly, different networks of reverse networks were trained, and the training volume was still set to 50 epochs. The result is shown in the right figure of Fig. 4. When the number of FC layers is 7 and the number of FE layers is 3, the network reaches the lowest MSE, which is about 0.1756.

We found that a larger number of network layers will produce a gradient explosion phenomenon, which will cause the network to fail to converge, and the loss is infinite, so it is not listed in the figure.

### Data preprocessing

In order to train a more reliable forward network, the reflectance data is re-divided, and it is stitched with the refractivity of Au and SiO_2_ corresponding to each frequency. The collated data is then normalized and input to the forward network, which can greatly improve the accuracy of the forward network.

In order to ensure that the data with larger values will not have a higher impact on the network than the data with smaller values, the input data needs to be normalized to make each column of data conforms with the standard normal distribution (the mean value is 0, the variance is 1), and then the processed data *x* can be expressed as follows:

1 $$ x=\frac{\left({x}_0\hbox{-} \mu \right)}{\sigma } $$

In the expression, *x*_0_ is the sample’s original data, *μ* the sample’s mean, and *σ* the sample’s standard deviation. If the input data is not re-divided, the reflectance will be distorted after normalization, which will reduce the accuracy of the network. The re-divided data will not affect its distribution due to normalization.

### Neural network method

The principle of the neural network is to build a lot of neurons (nodes) by imitating the way the human brain works and learns [27]. Neurons are connected with each other, and the output is adjusted by adjusting the connection weight. The output of the *j*th node of a layer can be expressed as follows:

2 $$ {y}_j=\frac{\sum \limits_{i=1}^nf\left({w}_i{x}_i+{b}_j\right)}{n} $$

*f* is the activation function, *w*_*i*_ is the connection weight of the previous layer’s *i*th node connected to the *j*th node, *x*_*i*_ is the output of the *i*th node of the previous layer, *b*_*j*_ is the bias term of this node, and *n* is the number of nodes in the previous layer connected to the *j*th node.

### Choice of an activation function

In order to meet the high nonlinearity of the inverse problem, the ELU function [28] is used as the activation function of each layer of neurons [28]. The output *f*(*x*) of the ELU function can be expressed as piecewise form as follows:

3 $$ f(x)=\left\{\begin{array}{c}x\\ {}\alpha \left({e}^x-1\right)\end{array}\right.{\displaystyle \begin{array}{c},\\ {},\end{array}}{\displaystyle \begin{array}{c}x\ge 0\\ {}x<0\end{array}} $$

In this function, *x* is the original input, and the parameter value for *α* ranges from 0 to 1.

**Fig. 5:**
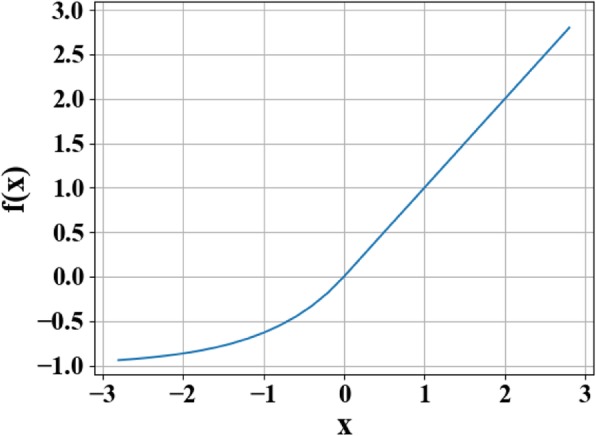
Exponential linear units (ELU) function curve. In the figure, *x* represents the original input, and *f*(*x*) represents the function output

The reason for using the activation function is that the activation function changes the nonlinear expression ability of each layer of the network, thereby improving the overall nonlinear fitting ability of the network. As shown in Fig. 5, the ELU function combines the advantages of Sigmoid and rectified linear unit (ReLU) activation functions. When *x* < 0, it has better soft saturation, which makes the network more robust to input changes and noise. When *x* > 0, there is no saturation, which is helpful to alleviate the disappearance of the gradient of the network. The feature that the mean value of ELU is close to 1 can make the network easier to fit. The result proves that using ELU as the activation function of the deep learning, neural network improves the robustness of the network significantly.

### Weight initialization scheme

The initialization method of the network weight of each layer determines the speed of the network fitting and even determines whether the network can fit or not. Variance scaling initialization is based on the amount of input data at each layer and extracts weights from a truncated normal distribution centered on 0, so that the variance can be reduced to a certain range, then the data can be spread deeper across the network [29]. On this network structure, using variance scaling initialization can make the network’s convergence speed significantly faster.

### Overfitting solution

Because of insufficient data, the network will produce some overfitting. With reduced overfitting, the network can have good generalization performance on data outside the training set. L2 regularization (also called weight-decay in regression problems) is used to process the weight *w*. The regularized output *L* can be expressed as follows:

4 $$ L={L}_0+\frac{\lambda }{2n}\sum {w}^2 $$

In Eq. (4), *L*_0_ represents the original loss function, and a regularization term $\frac{\lambda }{2n}\sum {w}^2$ is added on this basis, where *λ* represents the regularization coefficient, *n* the data throughput, and *w* the weight. After the regularization term is added, the value of the weight *w* tends to decrease overall, and the occurrence of excessive values can be avoided, so *w* is also called weight attenuation. L2 regularization can reduce the weight to avoid a large slope of the fitted curve, thereby effectively alleviating the overfitting phenomenon of the network and helping to converge.

On this basis, the dropout method is also used. This method can be visually regarded as “hiding” a certain scale of network nodes for each training, and hiding different nodes during each training, to achieve the goal of training multiple “partial networks”. And through training, most of the “partial networks” can accurately represent the targets, and the results of all the “partial networks” can be sorted to obtain the solution of the targets.

Using the L2 regularization and dropout methods mentioned above can not only effectively alleviate the low generalization caused by insufficient data, but also reduce the impact of a small amount of erroneous data in the data set on the training results.

On this network structure and data set, with dropout = 0.2 and L2 regularization coefficient *λ* = 0.0001, the network can obtain similar accuracy on the training set and test set, thereby achieving a high generalization performance.

## Result and discussion

**Fig. 6:**
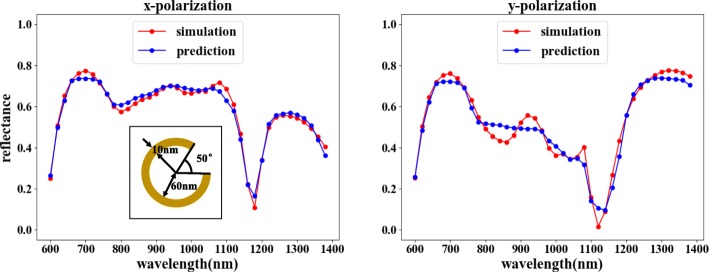
Results of forward network training. The corresponding structural parameters are *θ* = 50°, *r* = 60 nm, and *d* = 10 nm. In the figure, the horizontal axis represents the incident light wavelength, the vertical axis represents the reflectivity, the red line represents the COMSOL simulation result, and the blue line represents the network training result. The left figure shows the reflectivity curve corresponding to the *x*-polarized input, and the right figure shows the reflectivity curve corresponding to the *y*-polarized input

After training, our forward network can achieve a high degree of fitting, with an MSE of 0.0015, which shows the output is very similar to the simulation results, as shown in Fig. 6. This also ensures that when training the reverse network, the results of the reverse network can be reliably verified.

Finally, we will generate two models from the learned network and connect the two models to achieve the prediction function.

**Fig. 7:**
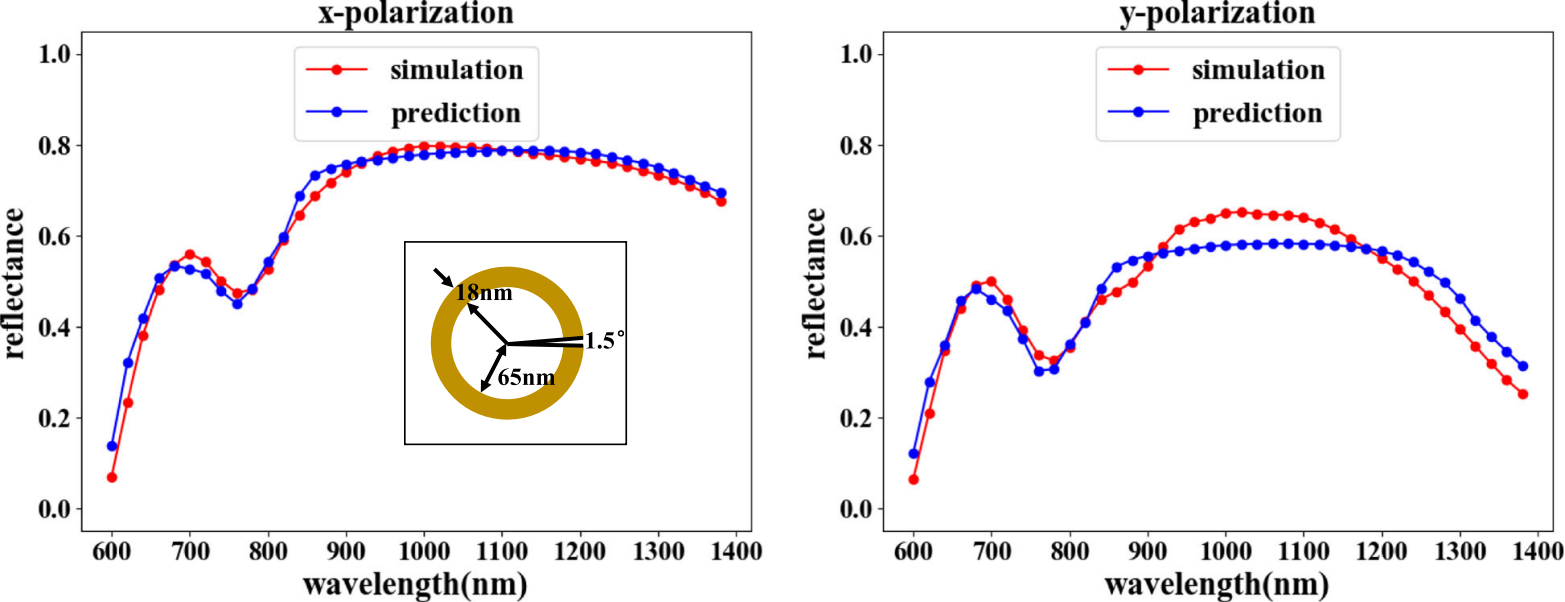
The reverse network followed by a forward network can achieve the purpose of prediction. In the figure, the horizontal axis represents the incident light wavelength, the vertical axis represents the reflectivity, the red line represents the COMSOL simulation result, and the blue line represents the network training result. The left figure shows the reflectivity curve corresponding to the *x*-polarized input, and the right figure shows the reflectivity curve corresponding to the *y*-polarized input. The predicted results for the input wavelength-reflectance curve are *θ* = 1.5°, *r* = 65 nm, and *d* = 18 nm

The prediction function can choose the combination shown in Fig. 2a. The reverse network predicts the corresponding structure according to the required wavelength-reflectance curve, and the forward network verifies the optical response of the structure. As shown in Fig. 7, by comparing the verified reflectance to the input reflectance, the reflectance characteristics of the incident light in the two polarization directions are basically consistent. Although minor reflectance mismatching is observable for certain wavelength values, the overall matching trend is clearly irrefutable, since the errors are well within an acceptable range.

## Conclusion

In this article, we have presented our designed deep learning network, capable of creating various effects through employing flexible combinations of network configurations. Our reverse network designed can predict the required structure using the input wavelength-refractive curve, which can greatly reduce the time required in solving the reverse problem and meet different needs through utilizing flexible combinations. The results indicate that the network has achieved a higher accuracy in predictions, which further implies that on-demand design can be solved through our method. Using deep learning to guide the design of metamaterials can automatically obtain more accurate metamaterial structures, a result unattainable by traditional design methods.

## Data Availability

The date the manuscript comes from our simulation network, and we cannot share it because of some personal reasons.

## Abbreviations

ELU: Exponential linear units
FC layer: Fully connected layer
FE layer: Feature extraction layer
FNN: Forward neural network
MSE: Mean square errors
ReLU: Rectified linear unit
RNN: Reverse neural network
SRR: Split-ring resonator
